# Supplementary motor area microstructure defines the extent of gait impairment in Parkinson’s disease

**DOI:** 10.1038/s41531-025-01119-4

**Published:** 2025-08-25

**Authors:** Paweł P. Wróbel, Annika Peter, Maja Kirsten, Alessandro Gulberti, Maxim Bester, Einar Goebell, Bastian Cheng, Yogesh Rathi, Ofer Pasternak, Tim Magnus, Götz Thomalla, Fanny Quandt, Robert Schulz, Focko L. Higgen, Monika Pötter-Nerger

**Affiliations:** 1 Department of Neurology, University Medical Center Hamburg-Eppendorf, Hamburg, Germany; 2 Department of Neuroradiology, University Medical Center Hamburg-Eppendorf, Hamburg, Germany; 3 Psychiatry Neuroimaging Laboratory, Brigham and Women’s Hospital, Harvard Medical School, Boston, USA; 4 Department of Radiology, Brigham and Women’s Hospital, Harvard Medical School, Boston, USA; 5 Department of Psychiatry and Psychotherapy, University Medical Center Hamburg-Eppendorf, Hamburg, Germany

**Keywords:** Neuroscience, Neurology

## Abstract

Gait disorders and freezing of gait are challenging symptoms in Parkinson’s disease. Cortical gait centers as the supplementary motor area appear to be relevant in gait control. We hypothesize that diffusion-MRI microstructural markers in this area are associated with quantitative gait performance in participants with Parkinson’s Disease. Retrospective clinical data showed that lower fractional anisotropy in the right supplementary motor cortex was associated with better performance in various bilateral quantitative gait parameters at normal speed, maximal velocity, and especially during cognitively demanding conditions as dual-tasking. Gait performance did not correlate with cortical thickness. In contrast, the correlation between gait and microstructure in the supplementary motor area could indicate that diffusion-MRI might function as a clinical biomarker. The added value might support the clinical prognosis and aid in the development of new interventions, such as localized and personalized stimulation techniques.

## Introduction

Gait impairment, particularly freezing of gait (FoG)^1^, significantly impacts the quality of life in Parkinson’s Disease (PD)^2^. However, its pathophysiology is still not well understood. Within the neuronal network involved in gait control, the supplementary motor area (SMA) has been identified as a relevant structure beneath the mesencephalic locomotor region for producing, modulating and transmitting the motor programs necessary for purposeful gait^3^, especially during increased cognitive load^4^. Anatomically, FoG studies^1,5^ indicated that the SMA is connected to the pedunculopontine nucleus (PPN) in the mesencephalic locomotor region through direct and indirect projections via the basal ganglia. The functional link between the SMA and the mesencephalic locomotor region has been demonstrated in participants with gait disorders^6^ and in white matter disconnection syndromes in both animals and humans^7–9^. SMA activity was also linked to the initiation and sequencing of voluntary movements^10,11^ in non-FoG individuals. Positron emission tomography studies revealed that SMA metabolism increases during gait in healthy adults^12,13^. In participants with gait impairment, the SMA metabolism is relatively decreased^14^. Of note, in fMRI studies BOLD signal from SMA was related to lower step length^15,16^. SMA pathology contributes to gait impairment seen in studies on ischemic stroke^17^ or corticectomy^18^. Therefore, the SMA appears to be an important cortical interface for cognitive gait control, and its structural alterations may serve as a relevant biomarker for gait disorders.

However, there are no clear results linking cortical atrophy topology to gait impairment^19–21^. A meta-analysis found no differences in cortical thickness (CT) between PD participants and controls, indicating that CT might not be a suitable measure for PD-related pathology^22^. On the other hand, it was discussed that the variability in CT findings in PD between studies may be related to the disease stage^23^. The heterogeneous results have led to increased demand for alternative approaches^24,25^, to measure surrogates of microstructural complexity. Diffusion-tensor imaging (DTI) was developed to provide insights into the microstructure of white matter^26^. New evidence demonstrates that DTI measures also reflect gray matter microstructure when compared to histological data^27,28^. For instance, axial diffusivity (AD) is sensitive to neuronal columns, and reflects the density of apical dendrites^29^. Radial diffusivity (RD) is sensitive to diffusion orthogonal to neuronal columns and correlates positively with the number of neuronal structures and thus possibly with the dendritic arborization^28,30^. Fractional anisotropy (FA), a scalar value that expresses the anisotropy of water diffusion, is positively influenced by AD and negatively influenced by RD. To date, an increase in cortical FA has been shown in aging^31^, cortical pruning^32^, and after concussion^33^, while lower FA was reported during maturation-associated dendritic spreading^34,35^.

Data on SMA activity in gait performance indicate a structure-gait relationship despite inconsistent reports on cortical thickness. Therefore, this retrospective work assesses the structure-gait relationship between SMA and gait performance. We hypothesized that the microstructural features of the SMA, as measured by DTI in a fMRI-predetermined region^36^, correlate with objective gait characteristics in PD participants. As PD is associated with compromised gait parameters^37^, further confirmation of SMA as a key structure in gait impairment would be of great therapeutic relevance, particularly for local stimulation, given the disability-driven gait impairment and its frequent resistance to treatment. Furthermore, we hypothesized that the structure-gait relationship would manifest especially in the examination of gait during cognitive load and fast walking performance.

## Results

### Behavioral Data

PD participants exhibited a heterogeneous extent of clinical axial symptom load as reflected in common motor scores. Based on MDS-UPDRS subitem 2.13 Freezing, 10 participants were classified as Freezers and 18 as Non-Freezers. In PD participants, the severity of general motor symptoms OFF medication was moderate, with Hoehn and Yahr stages 2-3 (median: 2). The mean MDS-UPDRS III score was 34.82 ± 10.54, and the mean Postural Instability and Gait Disorder (PIGD) Items 3.7-3.13 score was 9.68 ± 4.21. Axial symptoms were heterogeneous, with rater-based FoG Ziegler Scores averaging 7.22 ± 7.05. PD participants revealed impaired gait characteristics, with lower mean step lengths (left: 50.32 ± 11.86 cm; right: 52.61 ± 12.20 cm), higher mean step count (11.10 ± 3.23), reduced velocity (93.21 ± 23.96 cm/s), cadence (109.88 ± 12.36 steps/s), and extended stance time percentage of the step cycle (left: 66.24 ± 3.22%; right: 66.90 ± 3.22%), when compared to age matched healthy cohorts^37^. Among all 519 single walks analyzed (both OFF and ON medication), we identified only 2 episodes of brief akinetic freezing and 3 episodes of brief festinations, all occurring during OFF medication walks. Since FoG was such a rare event on the GaitRite carpet, we adopted a more comprehensive approach by evaluating interictal proxies of FoG, such as gait asymmetry and the coefficient of variation (CoV). Gait asymmetry (normal gait: 1.05, fast gait: 1.05, dual-task: 1.1) and CoV (normal gait: 3.78, fast gait: 4.10, dual-task: 9.47) were pathologically increased, especially during tasks with higher cognitive load (see Suppl. Table 2-4).

### Association between SMA microstructure and gait performance under normal walking conditions during quantitative gait analyses

**Fig. 1:**
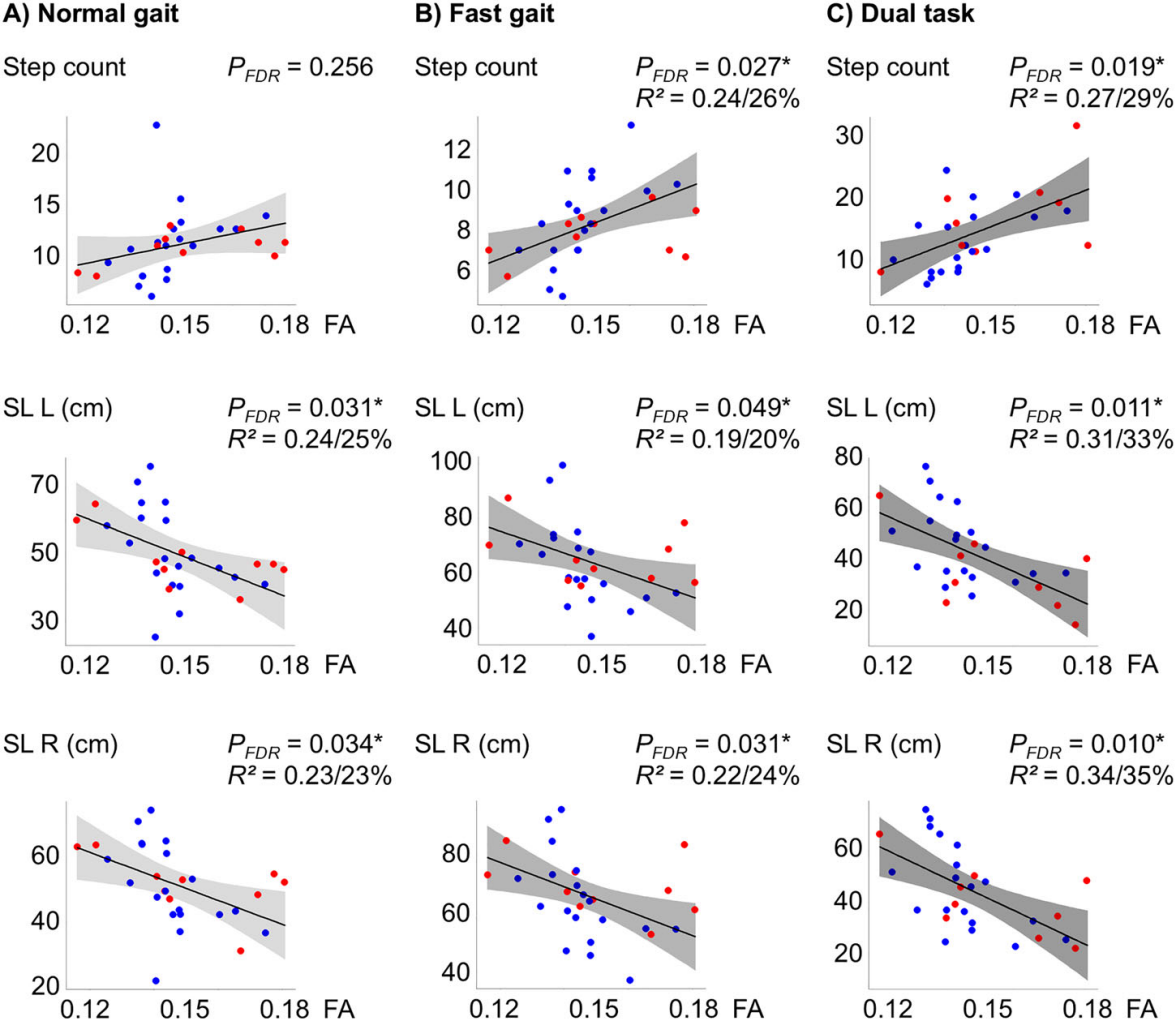
Relationship of right Supplementary Motor Area (SMA) anisotropy with step count and step length (SL). Fractional anisotropy (FA) values are plotted against step count and step length (SL) for the right (SL R) and left (SL L) leg under three conditions: **A** normal gait, **B** fast gait, and **C** dual-task. Dot-coloring: MDS UPDRS III FoG score ≥1: red, FoG score of 0: blue. Lower FA values, are associated with fewer and longer steps. Raw data points are presented along the model fit. *P*_*FDR*_ - *P*-value corrected for False-Discovery-Rate (FDR), *R²* - the partial *R²* value for FA from the model - the amount of unexplained variance reduced by the FA value is appended as a percentage.

**Fig. 2:**
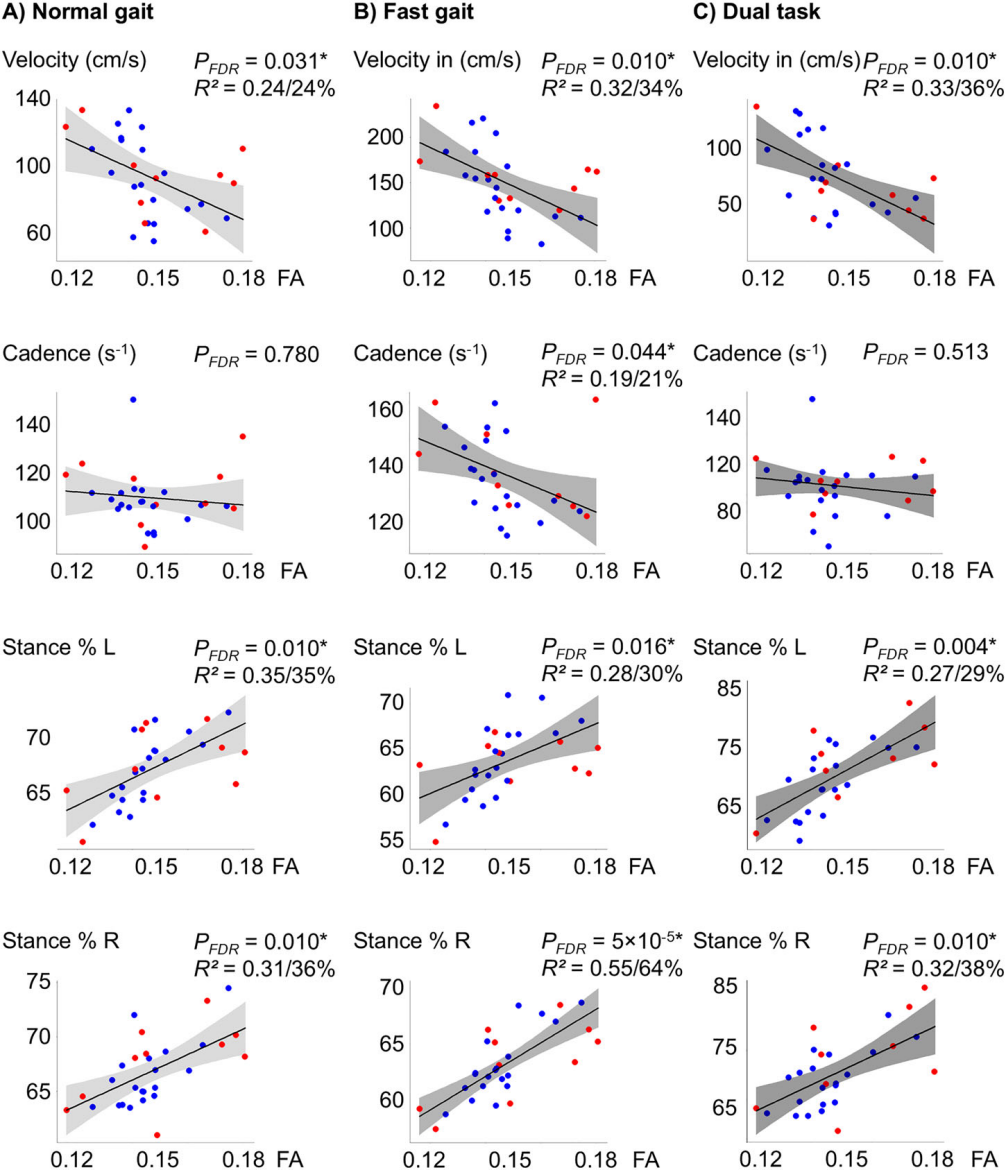
Relationship of right Supplementary Motor Area (SMA) anisotropy with velocity, cadence and stance time (% cycle). Fractional anisotropy (FA) values were plotted against gait velocity, cadence, and the percentage of stance time during the step cycle for each leg under three conditions: **A** normal gait, **B** fast gait, and **C** dual-task. Dot-coloring: MDS UPDRS III FoG score ≥1: red, FoG score of 0: blue. Lower FA values, indicative of greater microstructural complexity, are associated with faster gait velocity, higher cadence and shorter stance times. Raw data points are presented along the model fit. *P*_*FDR*_ - *P*-value corrected for False-Discovery-Rate (FDR), *R²* - the partial *R²* value for FA from the model - the amount of unexplained variance reduced by the FA value is appended as a percentage.

The associations between fractional anisotropy (FA) in the right SMA and gait performance during quantitative gait analyses under normal walking conditions at self-paced, regular speed are summarized in Figs. 1 and 2 (left columns). During normal gait, higher right SMA FA was associated with shorter step lengths of the left (*P*_*FDR=*_ 0.031) and right leg (*P*_*FDR=*_ 0.034), with lower gait velocity (*P*_*FDR=*_ 0.031) and longer percentage of stance time during the step cycle for left (*P*_*FDR=*_ 0.031) and right (*P*_*FDR=*_ 0.031) leg. Right SMA FA accounted for over 25% of the unexplained variance in these parameters. The results were reproduced using the leave-one-out-analysis (LOOA) approach. Right SMA FA was not significantly linked to the overall step count (*P*_*FDR=*_ 0.253) and cadence (*P*_*FDR=*_ 0.780). No significant associations were observed between FA in the left SMA and gait parameters after correction. Additionally, no significant associations were found between CT and quantitative gait parameters.

### Association between SMA microstructure and gait performance under increased cognitive load during quantitative gait analysis

Figures 1 and 2 display visualized plots of relationships between right SMA FA and fast (middle columns) and dual-task (right columns) gait performances. During fast gait, both legs exhibited a greater number of steps (*P*_*FDR*_ = 0.027) and reduced step length (left leg: *P*_*FDR*_ = 0.049; right leg: *P*_*FDR*_ = 0.031), which correlated with higher right SMA FA values.

The same pattern of a strong relationship between right SMA FA and gait measures was observed in dual-task gait conditions (Step count: *P*_*FDR=*_ 0.019; Step length left: *P*_*FDR=*_ 0.011, right *P*_*FDR=*_ 0.010). Higher right SMA FA correlated significantly in both fast and dual-task conditions with lower gait velocity (fast (*P*_*FDR=*_ 0.010), dual-task (*P*_*FDR=*_ 0.010)), and percentage of stance time (fast left leg (*P*_*FDR*_ = 0.016), fast right leg (*P*_*FDR*_ = 5 × 10^−5^), dual-task (left: *P*_*FDR*_ = 0.004, right: *P*_*FDR*_ = 0.010). Lower cadence was statistically linked to higher right SMA only in fast gait (*P*_*FDR=*_ 0.044). The right SMA FA values reduced the unexplained variance by 20–64%. The results were reproduced using the LOOA approach, except for the step length of the left leg and cadence in fast gait conditions, which exhibited a trend. An unadjusted supplementary analysis of the dual-task cost, which reflects the difference between normal and dual-task performance relative to normal performance, revealed significant correlations between right SMA FA and the dual-task cost across nearly all gait parameters, except for cadence. These findings, which underline the role of the right SMA during cognitively demanding gait tasks, are illustrated in Supplementary Fig. 1. Again, no significant associations were found for the FA in the left SMA.

### Relation of SMA microstructure to clinical Parkinsonian motor impairment scores

We evaluated whether clinical axial motor scores associated with MRI-derived measures of SMA microstructure. In summary, the correlation of SMA FA did not yield any significant results in any of the hemispheres in individuals with or without FoG. The right SMA FA was not correlated with MDS-UPDRS Part III general motor symptoms (*P* = 0.4324), Postural Instability and Gait Disorder (PIGD) subitems 3.7–3.13 (*P* = 0.9096), the FoG Ziegler Score (*P* = 0.1513), the levodopa equivalent dose (LEDD) (*P* = 0.6326) and the Montreal Cognitive Assessment Test (MoCA) scores (*P* = 0.1147) when adjusting for the cortical thickness. No relationship was observed between PIGD subitems 3.7-3.13 (*P* = 0.2680) and MoCA performance (*P* = 0.2219). The results are summarized in the Supplementary Fig. 1.

### Sensitivity analyses of other model input factors as gender, other cortical regions or scanner

Sensitivity analysis in sex-based subgroups (18 males, 11 females) replicated the significant findings in men for 15 of 21 tests (Normal gait: velocity [*P*_*=*_ 0.047], step length – left leg [*P*_*=*_ 0.046] and percentage of stance - left [*P*_*=*_ 0.012] and right [*P*_*=*_ 0.004] leg; Fast gait: velocity [*P*_*=*_ 0.015], step count [*P*_*=*_ 0.019], step length – right leg [*P*_*=*_ 0.033] and percentage of stance - left [*P*_*=*_ 0.046] and right [*P*_*=*_ 2.42 × 10^−5^] leg; Dual-task gait: velocity [*P*_*=*_ 0.004], step count [*P*_*=*_ 0.013], step length – left [*P*_*=*_ 0.005] and right leg [*P*_*=*_ 0.006] and percentage of stance - left [*P*_*=*_ 0.001] and right [*P*_*=*_ 0.001] leg). In the female group, significant results were found only in the fast condition for all gait parameters except the percentage of stance for the right leg (Fast gait: velocity [*P*_*=*_ 0.002], cadence [*P*_*=*_ 0.003], step count [*P*_*=*_ 0.049], step length – left [*P*_*=*_ 0.042] and right leg [*P*_*=*_ 0.042] and percentage of stance – left leg [*P*_*=*_ 0.010]). Results were not corrected for multiple comparisons due to the weaker power. To test whether the neighboring primary leg motor cortex influences the results, a post-hoc sensitivity analysis of the FA in the primary leg motor cortex and objective gait parameters did not reveal any significant relationships (*P* > 0.05). Post-hoc analysis on diffusivity data, i.e., axial and radial diffusivity, revealed no statistically significant results. A supplementary analysis revealed no significant effects of the skewed factor SCANNER, even at the uncorrected level.

## Discussion

This retrospective study analyzed clinical and MRI data to determine if quantitative gait measures related to the PD gait disorder and FoG correlate with microstructural surrogate markers in the SMA. Results showed a significant association between lower FA in the right SMA and longer steps, higher gait velocity, and a reduced stance fraction during the step cycle. In contrast, CT showed no correlation with quantitative gait measures.

**Fig. 3:**
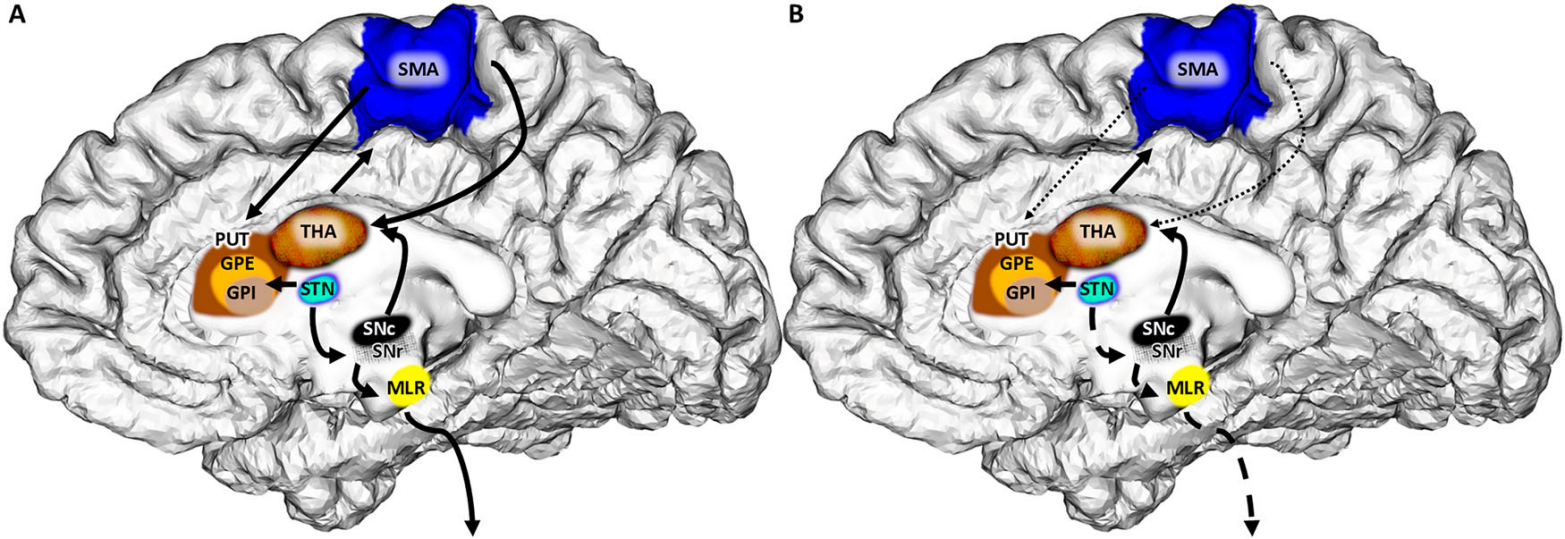
Influence of the right Supplementary Motor Area (SMA) on the pathophysiology of gait impairment). The lower fractional anisotropy (FA) of the right-hemispheric SMA in individuals with better gait performance could be interpreted as a sign of greater cortical complexity that aids in controlling gait. Compared to physiologic state (**A**), Parkinson’s Disease participants (**B**) with compromised SMA microstructure, display deteriorated control over the Striatum and Subthalamic nucleus, potentially facilitating gait impairment. THA: Thalamus, PUT: Putamen, GPE/I: Globus pallidus externus/internus. STN: Subthalamic nucleus. SNc/r: Substantia nigra, pars compacta/ reticularis, MLR: mesencephalic locomotor region. Not shown: the caudate nucleus.

The relationship between gait performance and the right SMA FA may reflect the established role of SMA activity in gait control^5,13,14^. The data suggest that microstructural, but not macroscopic measures of SMA, i.e., FA but not CT, are associated with its function within the neuronal gait control network. Higher FA in the right SMA of PD participants correlated with worse gait performance, which could be interpreted in different ways. It is important to note that the direction of FA changes in the gray matter is not necessarily the same as in the white matter. In comparative histology studies, FA was reported to decrease in dendritic spreading during cortical maturation^28,34,35^, and to increase with aging^31^ and during cortical pruning^32^. Lower FA might be a correlate of a more complex SMA in better-performing individuals. Earlier studies have shown a similar inverse relationship between cortical FA and behavioral performance^33^. A more complex histoarchitecture may enhance processing in SMA microcircuits, linking SMA activity to improved gait performance. Prospective studies should evaluate if additional white matter lesions might be a predictive or triggering component to impact gait in PD. Efficient local processing at the SMA may enhance gait-related macrocircuit modulation, leading to smoother gait (a conceptual representation is provided in Fig. 3).

Notably, the lack of significant findings for DTI measures in the primary leg motor area might suggest that the structure-function relationship for gait modulation may be particularly connected to the SMA. This could correspond to the indirect pathway of the gait model, involved in adapting gait to environmental changes between secondary motor regions and subcortical gray matter. Its dysfunction has been proposed to contribute to gait pathology^38^.

Histological studies dating back to Brodmann appear to support the link between cortical complexity and integrative specialization, indicating that integrative secondary heteromodal cortices have more interconnected structures than non-integrative primary unimodal areas^39,40^. Our earlier work on DTI and cortical specialization in younger and older subjects supports this hypothesis^41^. Of note, as also pointed out in our earlier work, cortical complexity does not necessarily equal a higher quantity of tissue. In neurodegenerative disease, a less prominent directionality within the cortex could also be considered a degeneration^42^. The lack of significant findings for AD and RD in this study could be linked to either a small sample size or heterogeneous processes detectable only by FA as a ratio measure. Taken together, one could suspect that the lower FA in better-performing PD participants could be interpreted as a sign of a more complex cortex^43^, leading to better performance given the preserved capacity. Still, these assumptions remain speculative, and further studies will be necessary to replicate the findings from this explorative work.

Numerically, the models for fast and dual-task gait characteristics and SMA microstructure frequently showed larger effect sizes than trials under normal gait conditions. This finding is supported by the replication of the findings in the dual-task cost analysis. If lower cortical anisotropy was linked to better performance, particularly during increased cognitive load, it could be a correlate of a cognitive load dependence of gait modulation by SMA. Behavioral studies suggest that gait impairment worsens in the presence of visual obstacles^4^. Existing stimulation studies indicate that the temporary impairment of the SMA influences the design of motor tasks that require greater focus given their complexity^44^. In the clinical setting, the suspected modulatory role of the SMA on gait in PD has already been postulated in combined PET/EEG studies^45^, especially in the right hemisphere. On a speculative note, it could be argued that microstructural complexity, as reflected in preserved interneuronal connections^34^, facilitates greater integrative capabilities or compensation. The numerically greater effect sizes of the relationships in cognitive load trials might support this assumption. However, it requires prospective studies in larger cohorts. A limitation might be the collinearity among the gait characteristics^46^, which could explain the observations within each single trial, however, we interpret the reproduction of similar findings within the three different, randomized gait tasks as a measure of reliability of the SMA-gait dependence at a qualitative level. On the other hand, the fast and dual-task conditions could probably lead to more varied results, thereby increasing sensitivity and thus the models’ power. Further, the data suggest differences between fast and dual-task data, for instance, in cadence, which could rely on different contributions from subcortical and cortical areas in processing. This ultimately requires interaction testing in a larger sample to facilitate discussion on a more robust basis. Both aspects of this retrospective patient analysis will require addressing in prospective studies with larger participant sizes.

Strikingly, the positive gait correlations were specifically associated with the right SMA, indicating possible cerebral hemispheric specialization. EEG studies show SMA involvement in externally paced bilateral foot movements^47^, their poor spatial resolution limits the analysis of both regions’ contributions. Functional MRI studies provide inconsistent results, with some showing greater activation in the right^16,48^ and others in the left SMA^15^ during gait-related imagery tasks. The small sample sizes and the fact that the tasks involved only mental imagery could be confounding factors in these studies. Early photon emission tomography studies, testing actual walking, found alterations in SMA metabolism on the right side (see Fig. 2. by Fukuyama et al.)^12^. When comparing the findings to macrostructural data, despite the lack of CT differences in PD participants reported in a meta-analysis, CT appears to decay particularly in the right but not left SMA^22^. The right-hemispheric finding in this study supports the CT data. Given the negative CT but positive DTI findings in this cohort, one could speculate that cortical DTI may be a more sensitive measure for detecting cortical pathology, as individuals in this work, who were candidates for deep brain stimulation surgery were not severely affected.

From a clinical perspective, this link might be relevant for application in early interventions as a preventive measure. The complex SMA could be discussed to be potentially a target for personalized interventions like right SMA cortical stimulation techniques. The SMA was repeatedly found as a relevant target for magnetic transcranial stimulation in gait impairment^49–51^. Given the limited effectiveness of pharmacologic treatments for gait impairments, alternative therapeutic approaches remain of great interest. Further, diffusion measures may identify individuals who could benefit more from stimulation techniques. Deep brain stimulation for gait impairments could be optimized by targeting the right SMA fiber bundles^52^. Finally, the enhanced sensitivity of DTI in detecting cortical pathology implies promising diagnostic applications at earlier disease stages.

Several limitations should be acknowledged and discussed. First, in terms of the design, the retrospective analysis was conducted without healthy controls, which needs to be addressed in future studies due to the lack of existing literature. Incorporating comparisons with healthy controls would enable a more detailed interpretation of the relationship between DTI and gait, offering a normative reference. Second, the study had a relatively small sample size. Therefore, we employed a conservative approach, incorporating the LOOA and corrections for multiple comparisons. Still, prospective studies with larger sample sizes are necessary. Third, SMA lateralization findings could be related to handedness. However, the high prevalence of right-handedness in this sample limits the ability to evaluate its effect. Fourth, the data were obtained from two scanners, which could have affected the results. However, the sensitivity analysis did not indicate a scanner effect. Additionally, diffusion weighting strength, indicated by b-values, could impact the results^53^. Therefore, further research will be necessary to assess the most suitable imaging parameters for neurodegenerative diseases. Lastly, the diffusion measures can be influenced by partial volume effects from white matter. The models were therefore adjusted for cortical thickness, accounting for the increased white matter signal in the thinner cortex.

This study is the first to connect microstructural properties of the right SMA to gait impairment in PD participants, highlighting its role in motor function planning and indicating a structure-gait relationship. Unlike the primary motor cortex, which showed negative findings, microstructural changes in the right SMA could help explaining bilateral gait control and its impairment in PD. The results indicate that future research at the microstructural level might be more likely to detect more subtle correlates for neural mechanisms, offering a point of origin for novel diagnostic and therapeutic approaches, to target symptoms of gait impairment.

## Methods

### Participants & clinical data

Data of idiopathic PD subjects were included if they underwent a standardized gait, clinical and MRI examination between July 2020 and October 2022. The Ethics Committee of the Medical Council of the State of Hamburg waived the Ethics Committee study approval, given the retrospective analysis and the consent for scientific evaluation given by the participants. The PD subjects provided written consent for the scientific evaluation of their clinical data. The MRI inclusion criterion required a dataset of diffusion-weighted data with 64 non-collinear gradients and T1-weighted data, both of which were collected for preoperative tractography before deep brain stimulation implantation. There were no subjects with drug-induced or genetic parkinsonism. The study included a total of 32 participants with PD. Datasets were excluded from the analysis if they showed insufficient image quality in the manual quality review by a neuroimaging-experienced physician. Following a manual quality assessment, three datasets were excluded due to impaired identifiability of the gray/white matter boundary. The final cohort comprised 29 participants (mean age 64.1 ± 6.93 years, 37.93% female, mean disease duration 10.93 ± 4.50 years). One participant’s dual-task data was not available, resulting in 28 datasets in the dual-task analyses. Detailed demographic and clinical information is provided in Tables 1 and 2. 12 PD participants revealed relevant gait disorders (MDS-UPDRS III item gait ≥2) and 10 PD participants revealed freezing of gait (FoG score ≥1). The imaging and clinical data acquisition was performed within 8 days, except for two individuals whose data were acquired with intervals of 10 (patient 21) and 6 (patient 26) months due to SARS-CoV-2 pandemic regulations.

**Table 1:** Demographic and clinical data

Patient	Age	Sex	Disease duration (years)	Scanner	Primarily affected side	FoG
1	75	male	12	A	left	no
2	69	female	9	B	left	yes
3	69	female	11	A	left	no
4	66	male	10	A	left	yes
5	72	female	11	A	left	no
6	68	female	19	B	left	no
7	63	male	10	A	left	yes
8	52	male	12	A	left	no
9	58	male	9	A	left	no
10	58	female	11	B	left	yes
11	69	male	14	B	left	yes
12	61	male	8	B	NA	yes
13	71	female	3	B	NA	no
14	65	male	15	B	NA	yes
15	66	female	14	A	right	yes
16	61	female	6	A	right	no
17	63	female	9	A	right	no
18	55	male	11	A	right	yes
19	55	male	9	A	right	no
20	75	male	17	A	right	no
21	70	male	9	A	right	yes
22	63	male	13	A	right	no
23	58	male	11	A	right	no
24	60	male	4	A	right	no
25	72	female	16	A	right	no
26	47	male	9	B	right	no
27	61	female	9	A	right	no
28	68	male	10	A	right	no
29	69	male	13	A	right	no

Age range (years): 47–75 (mean: 64.1, median: 65.0, standard deviation (SD): 6.93. Gender ratio (♂: ♀): 62%: 38%. Freezing of gait (FOG) ratio (yes:no): 36%: 64%. Scanner A: 3 Tesla Siemens Prisma Scanner (Siemens Healthineers, Erlangen, Germany). Scanner B: 3 Tesla Siemens Skyra Scanner (Siemens Healthineers, Erlangen, Germany). *NA*: not available.

**Table 2:** Clinical data on disease severity

Patient	MDS-UPDRS (Part III)	H&Y Stage	PIGD Sum Items 3.7–3.17	FoG Ziegler score	LEDD	MoCA
1	45	2	8	7	1834.8	23
2	28	2	6	9	2200	24
3	35	3	11	0	1050	28
4	40	3	8	0	1432.5	26
5	27	2	9	12	1350	26
6	33	3	12	0	1150	22
7	28	3	11	4	1600	28
8	26	3	11	14	1520	23
9	32	2	1	11	880	25
10	21	3	7	1	1225	24
11	62	2	11	11	885.6	15
12	22	3	NA	NA	1800	27
13	39	3	16	NA	1171.2	23
14	52	3	12	11	2022.5	26
15	46	2	9	11	942.4	29
16	42	2	11	0	1176.5	29
17	37	2	9	0	1350	25
18	59	3	16	23	1670	29
19	40	3	20	0	692.4	26
20	28	2	15	12	1025	25
21	35	3	9	22	1192.4	29
22	30	2	5	8	1410	24
23	31	2	14	0	1124.2	29
24	37	3	11	20	1787.5	24
25	29	2	7	8	1180	25
26	36	2	8	4	1253	30
27	21	2	7	0	1342.4	30
28	27	2	5	2	1287	29
29	22	2	2	5	1181.2	23

Examination performed in the off phase. MDS-UPDRS (MDS-Unified Parkinson’s Disease Rating Scale) Part III: 21–62 (mean: 34.83, median: 43.00, SD: 10.54) – rated by the same physician; Hoehn and Yahr Stage (H&Y): range 2–3, mode: 2 19%: 81%; Postural Instability and Gait Disorder (PIGD) Questionnaire Items 3.7-3.13 Score: 1–20 (mean: 9.68, median: 9.00, SD: 4.21), Ziegler Score: 0–23 (mean: 7.22, median: 7.00, SD: 7.05), Levodopa Equivalence Dose (LEDD): 1150–1520 (mean: 1335.7, median: 1253.0, SD: 353.4), MoCA (Montreal Cognitive Assessment): 15–30 (mean: 25.52, median: 25, SD: 3.19), *NA*: not available.

### Gait parameters

Gait parameters were measured and calculated using a GAITRite® walkway system (CIR Systems Inc., Franklin, USA). The GAITRite® system consists of a walkway with overall dimensions of 90 cm × 7 m × 3.2 mm. Each participant was tested in an off situation, i.e., the dopaminergic and agonistic treatment was paused 24 h prior, and they completed three different gait tasks in a randomized order, including: (1) preferred self-paced gait (normal), (2) maximum speed walking without running (fast), and (3) dual-task walking while counting backward in increments of 7 (dual-task). Every task was performed three times, and the averaged data were used for the analyses, including step count (absolute), step length (cm), velocity (cm/sec), cadence (steps/min), and the percentage of the stance of each leg within the gait cycle time. The data were corrected for leg length. We used the implemented GAITRite software algorithm for the detection of Fog episodes, which was then manually controlled by the video recordings. We calculated outliers (Z-score > 3) of gait velocity, step length, step time and cadence of the 519 recorded (OFF and ON medication) single walks to evaluate the impact of FoG episodes on mean gait measures but did not find Z-score outliers. Detailed data are summarized in Supplementary Tables 2-4.

### Cerebral Imaging Data – Acquisition and Processing

Magnetic resonance imaging (MRI) was conducted using either a 3Tesla Prisma Scanner [Scanner A] or a 3Tesla Skyra Scanner [Scanner B] (both Siemens Healthineers, Erlangen, Germany) with a 32-channel head coil. 3D T1-weighted imaging datasets were acquired with a magnetization-prepared rapid acquisition gradient-echo sequence (MPRAGE) (Scanner A and B: echo time [TE]: 2.46 ms, repetition time [TR]: 1900ms, 256 slices with a field of view (FOV): 256 × 160 mm, slice thickness 1 mm and an in-plane resolution of 0.94 × 0.94 mm). Diffusion MRI data were acquired with one b0 image and 64 non-collinear gradient directions with a b-value of 1500 s/mm² in a 128 × 104 mm FOV (Scanner A: 75 slices, TE: 82 ms, TR: 10700 ms and voxel size of 2 × 2 × 2 mm; Scanner B: 70 slices, TE: 82 ms, TR: 10900 ms and voxel size of 2 × 2 × 2 mm).

T1-weighted structural data were segmented and parcellated using the FreeSurfer-based *recon-all* tool (version 6.0.1) to facilitate the identification of gray matter^54^. The segmentation data were then registered to the individual pre-processed (DWI) data using advanced Normalization Tools (ANTs, version 2.3.4)^55^.

The mean CT of both SMA was calculated after registering the Human Motor Atlas Template (HMAT)^36^ data to individual segmentations using FreeSurfer’s *vol2surf* and subsequently collected via *mri_segstats*.

Eddy current correction was applied for the DWI data, followed by a T1 image-based EPI distortion, motion correction and brain extraction using MRtrix3 (3.0.2) and FSL (6.0.1)^56,57^. Free water (FW) correction, to account for the vicinity of cerebrospinal fluid, was conducted according to previously established methods^58^ by fitting a bi-tensor model with pre-set FW diffusivity using MATLAB (Version R2020a, The Mathworks, Natick, MA, USA)^59^. The FSL-based non-linear transformation was applied to SMA labels (from Montreal Neurological Institute (MNI) space to individual DTI space) using *flirt*/*fnirt* tools, including nearest-neighbor interpolation. The labels were multiplied with a cortex mask to exclude voxels from non-gray matter tissue that might have been produced during the transformation. Finally, for each SMA averaged FA, axial diffusivity (AD) and radial diffusivity (RD) values were derived from FW-corrected tensor eigenvalues using a custom-written MATLAB script (version R2020a, The Mathworks, Natick, MA, USA).

### Statistical analysis

Statistical analyses were performed using *R*^60^(version 4.0.2). Linear regression models examined the relationship between gait parameters, namely velocity, cadence, step count, step length, and stance time as a percentage of the cycle time (STp), as the independent and MRI-measures of SMA as dependent variables of interest. Given the lateralization of SMA function as well as missing data (>10%) on the most affected hemisphere, the analyses were conducted for the left and right hemispheres. Age and Sex were treated as nuisance variables to adjust for potential confounding effects. For DTI measures, the models were further adjusted for CT in the corresponding SMA, to account for the potential partial volume effects of white matter^41^. Separate models were calculated for the left and right SMA, and for the left and right leg in both step length and STp assessment. Multiple comparison correction using the False-Discovery-Rate (FDR) for all 3 conditions, all 7 gait parameters, and both hemispheres, i.e., 42 results in total, was applied with a significance level at P_*FDR*_ < 0.05. The models were cross-validated through leave-one-out analyses (LOOA).

As the findings might be attributable to other factors, further sensitivity analyses for the right SMA were conducted to assess the model robustness. Given the potential influence of the highly-skewed factor scanner on behavioral and diffusion data, the models were recalculated with an interaction term between diffusion and scanner variables. Furthermore, a relevant microstructure-function relationship with gait could also coexist in the leg-area of the primary motor cortex. To this end, the models were recalculated for the gait data imaging from the cortical region comprising the primary motor leg area, i.e., G_and_S_paracentral Destrieux atlas label. To account for the possibility of residual higher order sex-specific effects despite adjusting for sex, the analyses were repeated for sex-based sub-groups. Due to the sample size and to avoid overfitting, we did not include the primarily affected side or the disease duration as covariates, or apply a multivariate model for all factors. Lastly, to quantify the difference in performance between dual-task and normal gait, the models were recalculated for the dual-task cost, i.e., the difference between normal and dual-task gait normalized by the normal gait performance.

## Supplementary information

Supplementary Material

## Data Availability

The data used in the statistical analysis is available in the supplementary material. The remaining data are of clinical nature and therefore can not be made available.
