# Supplementary material for: Supplementary motor area microstructure defines the extent of gait impairment in Parkinson’s disease
Source: NPJ Parkinsons Dis. 2025 Aug 25;11:260. doi: 10.1038/s41531-025-01119-4 (PMC12379217; doi:10.1038/s41531-025-01119-4)
Supplement: Supplementary file 1 — Supplementary Material [file 41531_2025_1119_MOESM1_ESM.docx]

**Supplementary Material for**

**Supplementary motor area microstructure defines the extent of gait impairment in Parkinson’s disease**

Paweł P. Wróbel, MD^1*^, Annika Peter, MD^1^, Maja Kirsten, M. Sc.^1^, Alessandro Gulberti, PhD^1^, Maxim Bester, MD^2^, Einar Goebell, MD^2^, Bastian Cheng, MD^1^, Yogesh Rathi, PhD^3,4^, Ofer Pasternak, PhD^3,4^, Tim Magnus, MD^1^, Götz Thomalla, MD^1^, Fanny Quandt, MD^1^, Robert Schulz, MD^1^, Focko L. Higgen, MD^1,5#^, Monika Pötter-Nerger, MD^1#*^

^1^ Department of Neurology, University Medical Center Hamburg-Eppendorf, Hamburg, Germany

^2^ Department of Neuroradiology, University Medical Center Hamburg-Eppendorf, Hamburg, Germany

^3^ Psychiatry Neuroimaging Laboratory, Brigham and Women’s Hospital, Harvard Medical School, Boston, USA

^4^ Department of Radiology, Brigham and Women’s Hospital, Harvard Medical School, Boston, USA

^5^ Department of Psychiatry and Psychotherapy, University Medical Center Hamburg-Eppendorf, Hamburg, Germany

* - corresponding authors

Email: m.poetter-nerger@uke.de

Email: p.wrobel@uke.de

| **Patient** | FA SMA | | CT SMA | |
| --- | --- | --- | --- | --- |
|  | left | right | left | right |
| 1 | 0.1642862 | 0.14601372 | 2.610 | 2.566 |
| 2 | 0.17904964 | 0.14494878 | 2.167 | 2.354 |
| 3 | 0.13495703 | 0.12555593 | 2.480 | 2.568 |
| 4 | 0.1458998 | 0.12199413 | 3.034 | 2.611 |
| 5 | 0.14235042 | 0.14102942 | 2.016 | 2.143 |
| 6 | 0.15084422 | 0.16547967 | 1.892 | 2.05 |
| 7 | 0.14323078 | 0.14344405 | 1.806 | 1.962 |
| 8 | 0.14820392 | 0.1406603 | 2.318 | 2.305 |
| 9 | 0.13146909 | 0.15202564 | 2.453 | 2.499 |
| 10 | 0.14159164 | 0.14089973 | 2.880 | 2.732 |
| 11 | 0.16623066 | 0.18066512 | 2.221 | 2.11 |
| 12 | 0.1722203 | 0.17741975 | 2.366 | 2.081 |
| 13 | 0.14481969 | 0.1482711 | 2.684 | 2.511 |
| 14 | 0.15718997 | 0.16704188 | 2.639 | 2.829 |
| 15 | 0.14791669 | 0.14890814 | 2.574 | 2.376 |
| 16 | 0.14713605 | 0.14402974 | 2.709 | 2.8 |
| 17 | 0.14686765 | 0.14793883 | 2.551 | 2.337 |
| 18 | 0.1742654 | 0.11611313 | 2.327 | 2.534 |
| 19 | 0.15911999 | 0.13631441 | 2.628 | 2.711 |
| 20 | 0.14547843 | 0.14815956 | 2.510 | 2.635 |
| 21 | 0.16144833 | 0.17240795 | 2.657 | 2.278 |
| 22 | 0.16319863 | 0.13911486 | 2.192 | 2.462 |
| 23 | 0.13933717 | 0.13627936 | 2.275 | 2.406 |
| 24 | 0.15607941 | 0.17478669 | 2.433 | 2.568 |
| 25 | 0.1394691 | 0.13266487 | 2.351 | 2.111 |
| 26 | 0.1479108 | 0.16047555 | 2.763 | 3.025 |
| 27 | 0.1385086 | 0.14355531 | 2.438 | 2.541 |
| 28 | 0.13715492 | 0.1350731 | 2.665 | 2.584 |
| 29 | 0.1491434 | 0.14382502 | 2.147 | 2.224 |

**Supplementary Table 1 | Patient imaging data**.

Summary of the imaging data for each patient. FA: fractional anisotropy, SMA: supplementary motor area. CT: cortical thickness.

| **Patient** | **Step length - left** | | | **Step length - right** | | | **Step count** | | |
| --- | --- | --- | --- | --- | --- | --- | --- | --- | --- |
|  | N | F | D | N | F | D | N | F | D |
| 1 | 40.92 | 58.13 | 35.75 | 42.37 | 66.50 | 36.36 | 12.67 | 8 | 12.33 |
| 2 | 39.79 | 55.46 | 41.83 | 47.30 | 62.49 | 45.86 | 13 | 8.67 | 12.33 |
| 3 | 58.76 | 70.84 | 51.79 | 59.76 | 72.03 | 51.66 | 9.33 | 7 | 10 |
| 4 | 65.26 | 87.59 | 66.03 | 64.24 | 85.17 | 66.42 | 8 | 5.67 | 8 |
| 5 | 44.61 | 58.52 | 35.74 | 47.84 | 60.91 | 36.88 | 11.33 | 9.33 | 15.33 |
| 6 | 43.32 | 51.18 | 34.71 | 43.49 | 54.78 | 32.64 | 12.67 | 10 | 17 |
| 7 | 45.72 | 64.85 | 31.14 | 49.77 | 74.23 | 39.19 | 11.67 | 7.67 | 16 |
| 8 | 25.44 | 47.87 | 29.13 | 21.66 | 47.16 | 24.53 | 23 | 11 | 24.67 |
| 9 | 49.04 | 56.33 | 45.25 | 53.54 | 57.84 | 47.94 | 11 | 9 | 11.67 |
| 10 | 47.86 | 57.48 | 23.01 | 54.43 | 67.54 | 33.87 | 11 | 8.33 | 20 |
| 11 | 45.62 | 56.77 | 40.73 | 52.61 | 61.31 | 48.37 | 11.33 | 9 | 12.33 |
| 12 | 47.29 | 78.54 | 14.19 | 55.14 | 83.78 | 22.07 | 10 | 6.67 | 32 |
| 13 | 40.58 | 50.46 | 33.24 | 42.47 | 50.04 | 31.97 | 13.33 | 11 | 17 |
| 14 | 36.67 | 58.26 | 29.13 | 31.05 | 52.83 | 25.91 | 12.67 | 9.67 | 21 |
| 15 | 50.79 | 61.76 | 46.64 | 53.38 | 64.78 | 50.25 | 10.33 | 8.33 | 11.33 |
| 16 | 60.35 | 69.30 | 63.54 | 61.57 | 69.60 | 62.14 | 8.67 | 7 | 8.67 |
| 17 | 46.60 | 67.88 | 51.30 | 43.81 | 64.26 | 45.93 | 11.67 | 8.33 | 11.33 |
| 18 | 60.45 | 70.33 | NA | 63.68 | 73.28 | NA | 8.33 | 7 | NA |
| 19 | 65.61 | 72.94 | 71.79 | 64.47 | 73.46 | 72.39 | 8 | 7 | 7 |
| 20 | 32.30 | 37.11 | 25.68 | 37.07 | 45.54 | 29.08 | 15.67 | 10.67 | 20.33 |
| 21 | 47.25 | 68.98 | 21.89 | 48.65 | 67.98 | 34.60 | 11.33 | 7 | 19.33 |
| 22 | 76.53 | 99.60 | 65.41 | 75.22 | 95.88 | 66.44 | 6 | 4.67 | 8 |
| 23 | 61.03 | 74.24 | 55.78 | 64.77 | 84.90 | 69.41 | 8 | 6 | 8 |
| 24 | 41.25 | 52.96 | 34.96 | 36.69 | 54.61 | 25.42 | 14 | 10.33 | 18 |
| 25 | 53.57 | 66.98 | 37.36 | 52.34 | 62.50 | 36.87 | 10.67 | 8.33 | 15.67 |
| 26 | 46.03 | 46.15 | 31.23 | 42.44 | 37.07 | 22.79 | 12.67 | 13.33 | 20.67 |
| 27 | 48.91 | 57.86 | 48.51 | 49.84 | 58.59 | 49.42 | 11 | 9 | 10.33 |
| 28 | 71.88 | 94.12 | 77.75 | 71.64 | 92.52 | 76.04 | 7 | 5 | 6 |
| 29 | 65.81 | 75.17 | 50.16 | 65.49 | 74.82 | 54.48 | 7.67 | 7 | 8 |

**Supplementary Table 2 | Overview of step length and count data.**

Individual gait data for each of the 29 patients’ datasets. Rounded data are presented. N: normal gait condition, F: fast gait condition, D: dual task gait condition

| **Patient** | **Velocity (m/s)** | | | **Cadence (s^-1^)** | | |
| --- | --- | --- | --- | --- | --- | --- |
|  | N | F | D | N | F | D |
| 1 | 65.87 | 122.03 | 30.83 | 94.93 | 117.47 | 49.33 |
| 2 | 65.80 | 130.40 | 69.87 | 89.33 | 133.07 | 95.33 |
| 3 | 110.60 | 184.33 | 99.80 | 111.97 | 154.33 | 115.73 |
| 4 | 134.07 | 234.67 | 138.80 | 124.27 | 163.10 | 125.73 |
| 5 | 87.87 | 153.40 | 37.40 | 113.67 | 154.07 | 61.83 |
| 6 | 77.20 | 112.77 | 42.57 | 106.73 | 127.57 | 75.53 |
| 7 | 78.23 | 158.73 | 62.13 | 98.43 | 137.33 | 106.17 |
| 8 | 57.37 | 118.00 | 73.53 | 151.13 | 149.30 | 177.37 |
| 9 | 95.93 | 119.67 | 86.37 | 112.33 | 125.90 | 111.20 |
| 10 | 100.83 | 158.43 | 36.53 | 117.93 | 151.60 | 76.90 |
| 11 | 110.83 | 162.27 | 73.77 | 135.57 | 164.20 | 97.10 |
| 12 | 90.00 | 164.57 | 36.97 | 105.40 | 121.97 | 123.80 |
| 13 | 65.33 | 96.30 | 41.17 | 94.23 | 114.87 | 75.53 |
| 14 | 60.80 | 119.73 | 58.33 | 107.57 | 129.27 | 127.20 |
| 15 | 92.97 | 132.93 | 85.47 | 107.00 | 125.97 | 105.57 |
| 16 | 110.07 | 144.47 | 119.10 | 108.27 | 124.77 | 113.83 |
| 17 | 79.93 | 168.07 | 82.90 | 106.33 | 152.80 | 101.77 |
| 18 | 123.93 | 173.43 | NA | 119.63 | 144.47 | NA |
| 19 | 116.00 | 154.73 | 132.27 | 107.03 | 126.87 | 110.03 |
| 20 | 55.03 | 88.67 | 42.63 | 95.40 | 129.17 | 93.33 |
| 21 | 94.90 | 143.53 | 44.40 | 118.70 | 125.53 | 89.20 |
| 22 | 133.93 | 221.07 | 117.83 | 105.90 | 135.47 | 107.00 |
| 23 | 117.37 | 183.93 | 113.47 | 111.97 | 138.67 | 106.20 |
| 24 | 68.90 | 111.30 | 55.80 | 106.40 | 123.77 | 109.90 |
| 25 | 96.30 | 158.17 | 58.10 | 109.17 | 146.87 | 93.10 |
| 26 | 74.37 | 82.50 | 49.87 | 100.90 | 119.50 | 110.87 |
| 27 | 89.00 | 133.13 | 72.87 | 108.23 | 137.20 | 89.27 |
| 28 | 125.83 | 216.27 | 134.60 | 105.17 | 139.17 | 104.97 |
| 29 | 123.77 | 204.77 | 85.67 | 113.03 | 162.83 | 98.23 |

**Supplementary Table 3 | Overview of gait velocity and cadence.**

Individual gait data for each of the 29 patients’ datasets. Rounded data are presented. N: normal gait condition, F: fast gait condition, D: dual task gait condition.

| **Patient** | **Stance % left** | | | **Stance % right** | | |
| --- | --- | --- | --- | --- | --- | --- |
|  | N | F | D | N | F | D |
| 1 | 68.20 | 64.43 | 76.37 | 68.00 | 61.87 | 73.80 |
| 2 | 71.40 | 64.53 | 71.07 | 68.43 | 63.10 | 68.93 |
| 3 | 62.07 | 56.60 | 62.57 | 63.57 | 58.70 | 64.10 |
| 4 | 60.50 | 54.70 | 60.30 | 64.53 | 57.37 | 64.93 |
| 5 | 66.87 | 62.03 | 73.23 | 65.33 | 62.07 | 74.60 |
| 6 | 69.40 | 66.70 | 75.03 | 69.23 | 67.00 | 80.37 |
| 7 | 70.80 | 66.83 | 74.00 | 70.43 | 65.13 | 73.80 |
| 8 | 70.80 | 67.17 | 71.30 | 72.00 | 65.23 | 71.50 |
| 9 | 68.03 | 66.60 | 68.63 | 68.63 | 68.43 | 70.50 |
| 10 | 67.17 | 65.30 | 77.97 | 68.07 | 66.27 | 78.27 |
| 11 | 68.70 | 65.10 | 72.17 | 68.17 | 65.20 | 70.97 |
| 12 | 65.80 | 62.27 | 78.53 | 70.17 | 66.30 | 84.90 |
| 13 | 68.83 | 66.50 | 71.93 | 66.93 | 63.87 | 69.03 |
| 14 | 71.77 | 65.77 | 73.20 | 73.30 | 68.50 | 75.20 |
| 15 | 64.57 | 61.40 | 66.53 | 60.97 | 59.67 | 61.17 |
| 16 | 65.00 | 62.87 | 63.37 | 64.97 | 62.80 | 65.53 |
| 17 | 68.87 | 61.47 | 67.83 | 64.57 | 61.23 | 65.87 |
| 18 | 65.20 | 63.20 | NA | 63.27 | 59.20 | NA |
| 19 | 64.33 | 62.10 | 62.10 | 63.77 | 62.40 | 66.00 |
| 20 | 71.70 | 70.93 | 75.67 | 65.33 | 62.17 | 68.70 |
| 21 | 69.13 | 62.80 | 82.73 | 69.30 | 63.37 | 81.70 |
| 22 | 62.77 | 58.63 | 64.00 | 63.50 | 61.23 | 63.63 |
| 23 | 65.50 | 62.67 | 59.03 | 67.37 | 62.30 | 70.80 |
| 24 | 72.37 | 68.07 | 75.13 | 74.47 | 68.70 | 76.73 |
| 25 | 64.73 | 59.33 | 69.57 | 66.03 | 61.07 | 70.00 |
| 26 | 70.60 | 70.63 | 76.80 | 66.90 | 67.70 | 74.20 |
| 27 | 67.20 | 64.70 | 67.83 | 65.00 | 62.67 | 64.43 |
| 28 | 63.20 | 60.50 | 62.33 | 63.70 | 59.93 | 63.63 |
| 29 | 64.33 | 59.57 | 67.87 | 64.17 | 59.47 | 68.30 |

**Supplementary Table 4 | Overview of each leg’s percentage of stance during the step cycle.**

Individual gait data for each of the 29 patients’ datasets. Rounded data are presented. N: normal gait condition, F: fast gait condition, D: dual task gait condition.


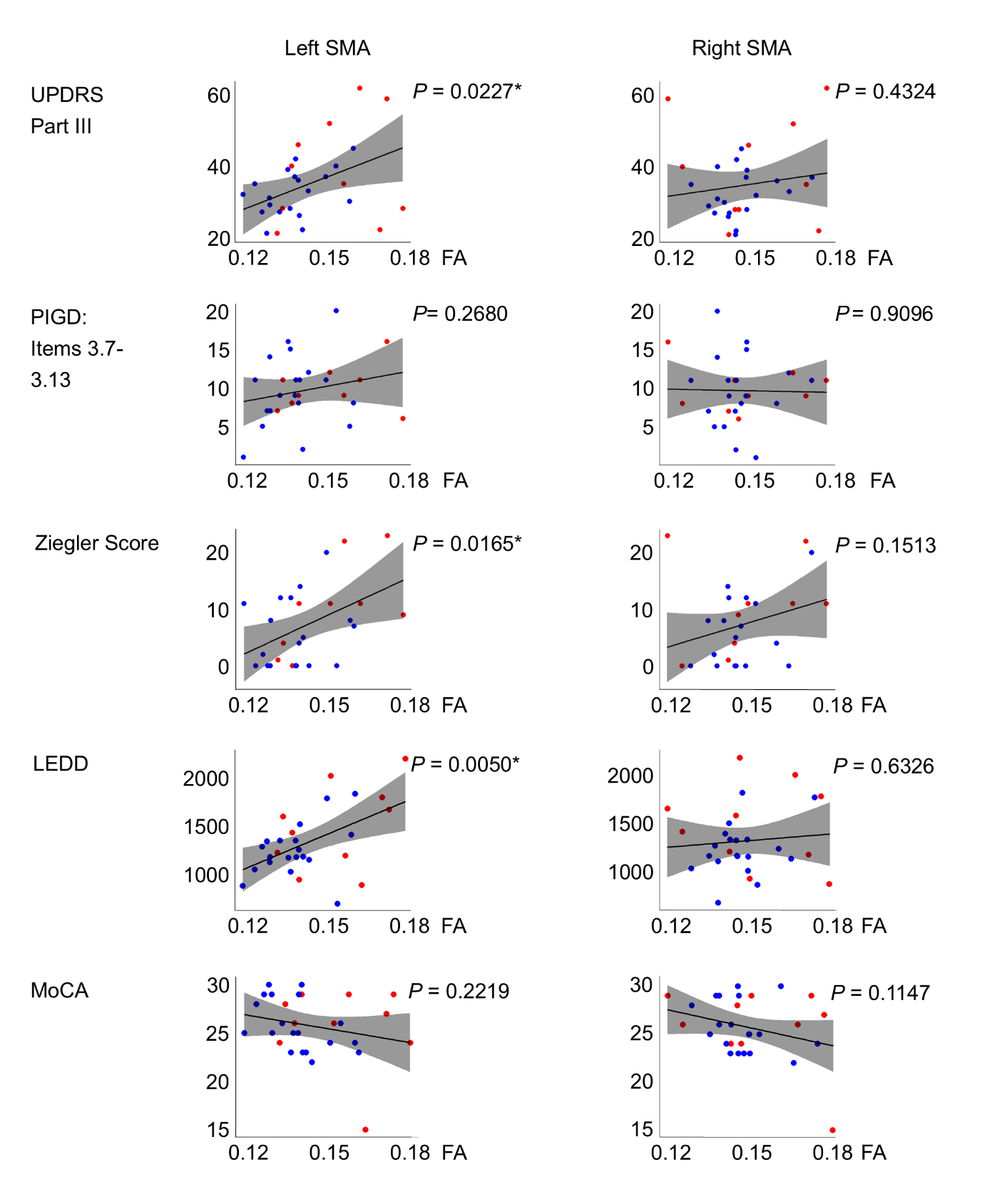


**Supplementary Figure 1 | Relationship of left and right Supplementary Motor Area (SMA) anisotropy with clinical and behavioral data.**

Fractional anisotropy (FA) values were plotted against the Unified Parkinson Disease Rating Scale (UPDRS) – examined in OFF, Postural Instability and Gait Disorder (PIGD) Questionnaire and the Ziegler Score – examined in OFF. LEDD: Levodopa equivalent dose. MoCA: Montreal Cognitive Assessment Test. Dot-coloring: MDS UPDRS III FoG score ≥1: red, FoG score of 0: blue. Raw data points are presented along the model fit. Uncorrected P-values are presented.


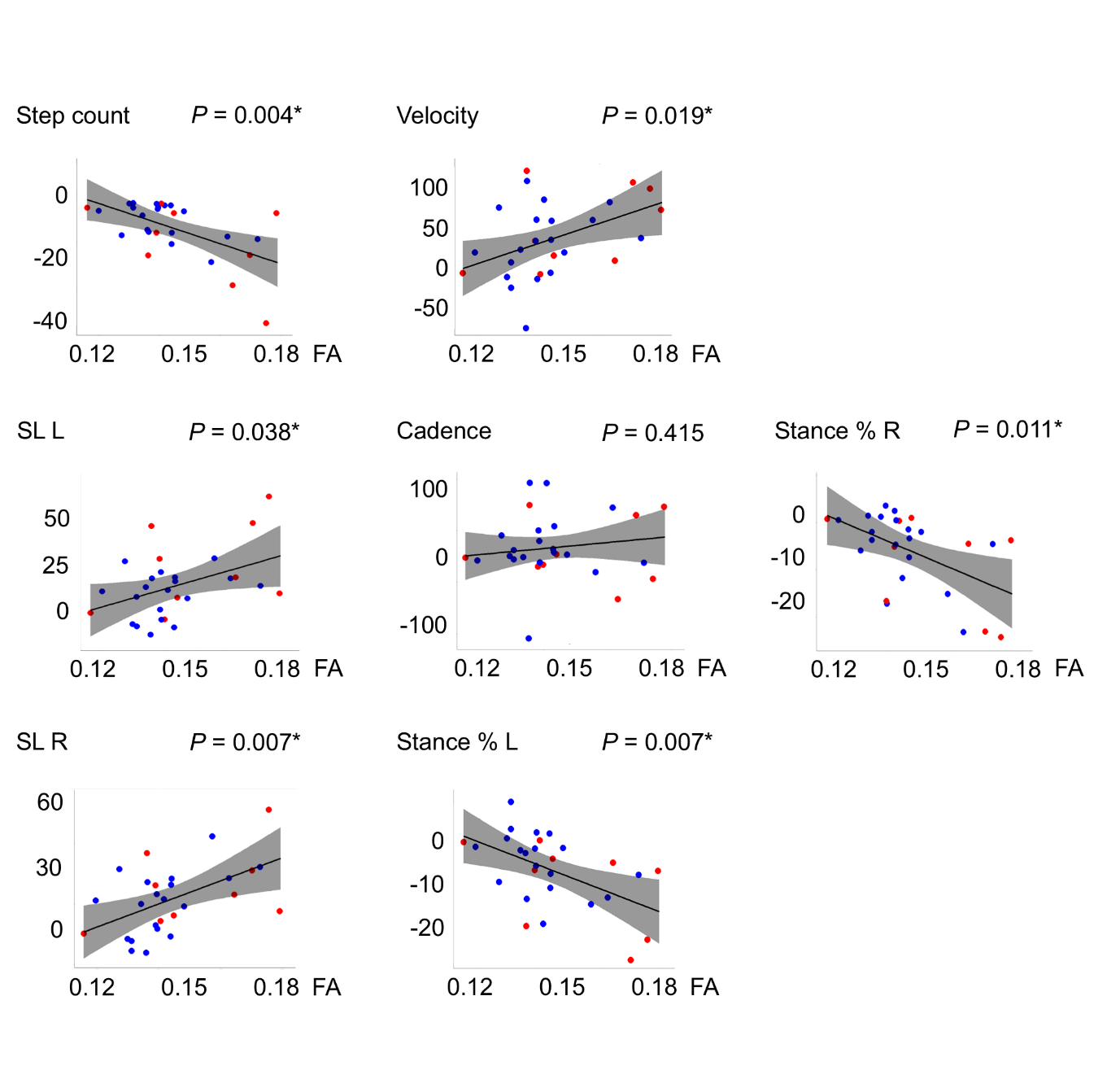


**Supplementary Figure 2 | Relationship of right Supplementary Motor Area (SMA) anisotropy with the dual-task cost of gait characteristics.**

Fractional anisotropy (FA) values were plotted against the dual-task cost of step count, step length (separate for each leg), gait velocity, cadence, and the percentage of stance time during the step cycle for each leg under three conditions. Dot-coloring: MDS UPDRS III FoG score ≥1: red, FoG score of 0: blue. Dual-task cost = ((Normal performance – Dual-task performance) / Normal performance) × 100. Lower FA values, indicative of greater microstructural complexity, are associated with greater dual-task costs for all gait parameters except cadence. Raw data points are presented along the model fit. Uncorrected P-values are presented. SL: step length, L: left, R: right.
